# A Comprehensive Analysis of Authorship in Radiology Journals

**DOI:** 10.1371/journal.pone.0139005

**Published:** 2015-09-25

**Authors:** Wilfred Dang, Matthew D. F. McInnes, Ania Z. Kielar, Jiho Hong

**Affiliations:** 1 Faculty of Medicine, University of Ottawa, Ottawa, Ontario, Canada; 2 The Ottawa Hospital Research Institute Clinical Epidemiology Program, Ottawa, Ontario, Canada; 3 The Ottawa Hospital Research Institute Cancer Therapeutics Program, Ottawa, Ontario, Canada; 4 Department of Radiology, University of Ottawa, Ottawa, Ontario, Canada; 5 Schulich School of Medicine and Dentistry, Western University, London, Ontario, Canada; Johns Hopkins Bloomberg School of Public Health, UNITED STATES

## Abstract

**Objectives:**

The purpose of our study was to investigate authorship trends in radiology journals, and whether International Committee of Medical Journal Editors (ICMJE) recommendations have had an impact on these trends. A secondary objective was to explore other variables associated with authorship trends.

**Methods:**

A retrospective, bibliometric analysis of 49 clinical radiology journals published from 1946–2013 was conducted. The following data was exported from MEDLINE (1946 to May 2014) for each article: authors’ full name, year of publication, primary author institution information, language of publication and publication type. Microsoft Excel Visual Basics for Applications scripts were programmed to categorize extracted data. Statistical analysis was performed to determine the overall mean number of authors per article over time, impact of ICMJE guidelines, authorship frequency per journal, country of origin, article type and language of publication.

**Results:**

216,271 articles from 1946–2013 were included. A univariate analysis of the mean authorship frequency per year of all articles yielded a linear relationship between time and authorship frequency. The mean number of authors per article in 1946 (1.42) was found to have increased consistently by 0.07 authors/ article per year (R² = 0.9728, P<0.001) to 5.79 authors/article in 2013. ICMJE guideline dissemination did not have an impact on this rise in authorship frequency. There was considerable variability in mean authors per article and change over time between journals, country of origin, language of publication and article type.

**Conclusion:**

Overall authorship for 49 radiology journals across 68 years has increased markedly with no demonstrated impact from ICMJE guidelines. A higher number of authors per article was seen in articles from: higher impact journals, European and Asian countries, original research type, and those journals who explicitly endorse the ICMJE guidelines.

## Introduction

The assignment of authorship credit when it may not be appropriate is a frequently reported problem in biomedical journals [1–5], and also encountered in radiology journals [6–9]. Several studies have noted increasing trends of inappropriate authorship in radiology over several decades. Chew (1988) observed increasing co-authorship and ‘fraudulent’ authorship in radiology journals. In a survey by Slone (1996) of authors from the *American Journal of Roentgenology*, the frequency of ‘undeserved’ authorship increased with increasing number of authors per article. Hwang (2003) observed that only 68% of authors published in *Radiology* fulfilled The International Committee of Medical Journal Editors (ICJME) guidelines for authorship [8, 10–12]. The reasons for the increases in authorship frequency are likely multifactorial such as research complexity (multicenter trials) and career advancement/ financially related factors. Honorary authorship, or authorship granted to individuals who did not make the necessary contributions, inappropriately give credit to these non-contributing authors and dilutes recognition from authors who did meet authorship requirements [4]. Institutional pressures to publish are often cited as causative factors for undeserving authorship. Authorship is highly correlated to: academic relevance, department funding, and career advancement [13–16].

The ICMJE sets guidelines regarding authorship to provide clear direction regarding authorship requirements and to address concerns surrounding undeserving authorship. These recommendations were released initially in 1979 and major revisions were issued in 1997, 2003, 2010 and 2013 [17]. Many medical journals have endorsed these guidelines to ensure proper attribution of authorship through enforcement of various authorship limitation policies, including disclosure forms, published disclosure statements, and, in some cases, limits to the number of authors per article [18–22].

Few large-scale studies have studied the effect of authorship limitation policies on the pattern of authorship [19, 23–28]. An objective and comprehensive evaluation of the impact of ICMJE guidelines on authorship in radiology journals has also not yet been done [9, 28].

The purpose of our study was to investigate trends in authorship in radiology journals, and whether ICMJE recommendations have had an impact on these trends. A secondary objective was to explore other variables associated with authorship trends.

## Materials and Methods

Research ethics board approval at our institution is not required for this study type.

### Data Retrieval

A list of 49 ‘clinical radiology journals’ (S1 Table) was comprised and organized by radiology topic of focus (S2 Table) by a fellowship trained radiologist (MM with 10 years of experience in imaging research) for analysis. The definition used for a ‘clinical radiology journal’ is one that publishes studies pertaining to medical imaging at the clinical level. Journals specifically focused on Nuclear Medicine and experimental techniques were excluded. The journals selected represent a wide range of clinical radiology topics and is varied in impact factor and history of publication.

Article data for each journal was retrieved and categorized from Ovid MEDLINE(R) In-Process & Other Non-Indexed Citations and Ovid MEDLINE(R) 1946 to May 2014 (2014 Ovid Technologies, New York, NY). Journals were limited by publication year from the first full calendar year each journal was indexed, for all available years of MEDLINE (from 1946–2013). Articles were also limited by publication type (see S2 Table for all publication types available) to exclude letters to the editor and editorials. The inclusion list of publication types was reviewed and selected by the senior radiologist (MM). Articles included must have had at least one main author indexed on MEDLINE. For research groups, each contributing author within the group must be listed.

The following data was exported from MEDLINE for each article: authors’ full name, year of publication, primary author institution information, language of publication and publication type. Data was entered on a Microsoft Excel spreadsheet (Microsoft Corporation, Redmond, Washington) for analysis.

The presence of ICMJE recommendations, or any documented authorship contribution policies published on each journal’s website was retrieved by JH, a 4^th^ year undergraduate student and reviewed by WD, a third year medical student. The authorship guidelines available on each journal’s website were categorized into the following: 1) Journal expresses explicit endorsement of ICMJE authorship guidelines. 2) Journal does not explicitly endorse ICMJE guidelines but has a statement regarding authorship criteria in their instructions available to authors. 3) Journal has no mention of ICJME guidelines or authorship contribution criteria on their website. In addition, journals that had no public access to their authorship criteria and required “an invitation” by the editorial committee were documented as having no explicitly stated authorship criteria.

The 2013 Impact Factor for each journal was obtained through Thomson Reuters: Journal Citation Reports [29].

### Data Analysis

A series of Microsoft Excel scripts and formulas were used to a) count the number of authors per publication and to categorize article data by b) country of origin of the primary author’s home institution, c) language of the article, and d) publication type.

Each indexed full author name was counted per article using Excel scripting.

Indexed institution information of primary authors was used to determine the primary author’s country of origin. For multi-institutional studies, the primary author’s first listed institution was used as their country of origin. Analyses of articles were completed from 1987 onwards, as author institutions were not indexed on MEDLINE by journals prior to this date.

Four major languages of publication were identified from 1946–2013: Italian, French, German and English. Any other language of publication was classified as “Other”.

Based on MEDLINE defined “Publication Type” categories, all articles were categorized into “Original Research”, “Review” or “Case Report” publication types. Reviews were defined as “Systematic Review” and “Review”, “Case Report” as “Case Reports” and “Original Research” as all remaining publication types selected. A manual analysis of the most recent year (2013) of all included radiology journals (WD) identified that 4.7% (423/7216) of Original Research articles could be further classified into different study types (e.g. Clinical Trial, Observational Study, Meta-analysis, Randomized Control Trial, Technical Report and Practice Guideline). Meta-analyses that were classified by MEDLINE as “Review” and “Meta-analysis” was classified into the “Review” category. Meta-analyses that could not be classified as a “Review” article was then classified as “Original Research”.

Following all computerized data analyses by Excel, two authors (WD and JH) independently reviewed the 3 most recent years of all included radiology journals for coding accuracy (2011–2013). Both authors agreed that coding was accurate for 100% (26,268/26,268) of these journal articles, κ = 1.

### Statistical Analysis

The overall mean number of authors per article, mean number of authors per article/year, as well as for each individual journal, country of origin, language, and publication type was assessed from 1946–2013.

These variables were calculated as follows:

$$\text{Overall mean number of authors}/\text{article}=\;\frac{\text{Total Authors by all Journals from }1946-2013}{\text{Total Articles by all Journals from }1946-2013}$$

$$\text{Mean number of authors}/\text{article per Year }=\;\frac{\text{Total Authors by all Journals per Year }}{\text{Total Articles by all Journals per Year}}$$

$$\text{Mean number of authors}/\text{article per Journal per Year}=\;\frac{\text{Total Authors in each Journal per Year}}{\text{Total Articles in each Journal per Year}}$$

$$\text{Mean number of authors}/\text{article per Country per Year}=\;\frac{\text{Total Authors each Country per Year}}{\text{Total Articles each Country per Year}}$$

$$\text{Mean number of authors}/\text{article per Language per Year}=\;\frac{\text{Total Authors per Language per Year}}{\text{Total Articles per Language per Year}}$$

$$\text{Mean number of authors}/\text{article per Article Type per Year}=\;\frac{\text{Total Authors each Article Type per Year}}{\text{Total Articles each Article Type per Year}}$$

Univariate regression analyses were used to investigate the correlation between the mean number of authors/article and time, as well as the authorship trends over time. This analysis was completed for mean number of authors/article per year, journal, country, language and publication type. All statistical analyses were performed using SPSS version 21.0 (IBM Corp. Released 2012. IBM SPSS Statistics for Windows, Version 21.0. Armonk, NY: IBM Corp). The change in mean number of authors/article per year was compared between radiology journal subjects/sub-categories using a Kruskal-Wallis Test. The effect of authorship contribution guidelines for journals published in 2013 was assessed using a Dunn’s Test for journals that follow ICMJE recommendations, journals that have policies on authorship contribution and articles that did not have any mention of authorship contribution instructions. Student t-tests were used to compare the average authorship before and after implementation and revision years of ICJME guidelines. Impact factor and average authorship for each journal in the 2013 year was also analysed for association using a Pearson’s Correlation test.

## Results

Table 1 (below) summarizes data for each journal analyzed. This includes number of articles and articles analyzed, mean number of authors/article, the change in number of authors/article per year over time and each journal’s categorization of ICJME endorsement.

**Table 1:** Per Journal Demographics and Linear Regression Analysis of Authorship over Time. Data collected per journal (Starting year in which data was collected on MEDLINE, total # of articles analyzed, total # of authors analyzed, mean number of authors/article for all years included per journal). Linear regression analysis of the relationship between mean number of authors/article per journal and time. See S1 Table for full journal names. Explicit endorsement of ICJME guidelines on each journal’s website was determined. 1) Explicitly endorses ICMJE. 2) Does not explicitly endorse ICMJE but has a statement regarding authorship criteria in their instructions for authors. 3) Has no mention of ICJME or authorship contributions.

Journal	Starting Year Included	Total Articles	Total Authors	Mean # of Authors/Article	Coefficient	CI (Lower)	CI (Upper)	P-Value	ICJME Endorsement
Abdominal Imaging	1994	2502	12237	4.89	0.092	0.074	0.11	<0.001	2
Academic Radiology	1995	3708	18119	4.89	0.161	0.114	0.209	<0.0010	2
Acta Radiologica	1988	3716	18658	5.02	0.086	0.061	0.109	<0.0010	1
AJNR	1981	9352	48453	5.18	0.084	0.073	0.094	<0.0010	1
AJR	1976	21130	92026	4.36	0.063	0.054	0.071	<0.0010	1
Brit. Journal of Radiology	1946	13195	41853	3.17	0.062	0.057	0.066	<0.0010	1
Can Assoc Radiol J	1986	1673	6046	3.61	0.046	0.009	0.083	0.016	1
Cancer Imaging	2005	479	1814	3.79	0.384	0.252	0.516	<0.0010	1
Clin Imaging	1990	1941	9630	4.96	0.095	0.076	0.114	<0.0010	2
Clin Radiology	1960	7088	24796	3.50	0.066	0.06	0.072	<0.0010	1
Eur J Radiol	1982	5938	31782	5.35	0.088	0.069	0.107	<0.0010	1
Eur Radiol	1996	6338	37316	5.89	0.213	0.179	0.247	<0.0010	1
Int J Cardiovas Imag	2002	1419	9371	6.60	0.172	0.07	0.273	0.004	2
Invest Radiol	1966	5725	27172	4.75	0.126	0.113	0.139	<0.0010	1
J Cardiovasc Magn Res	2000	850	5954	7.01	0.213	0.151	0.275	<0.0010	1
J Clin Ultrasound	1974	4029	16623	4.13	0.069	0.062	0.076	<0.0010	2
J Comput Assist Tomo	1978	6989	34103	4.88	0.081	0.075	0.087	<0.0010	1
J Digit Imaging	1989	1626	6718	4.13	0.049	0.02	0.078	0.002	2
J Magn Reson Imaging	1991	5606	32490	5.80	0.101	0.089	0.113	<0.0010	1
J Neuroimaging	1992	1376	6890	5.01	0.088	0.058	0.119	<0.0010	3
J Neuroradiol	1977	1244	6561	5.27	0.068	0.051	0.085	<0.0010	1
J Radiol Case Report	2008	360	1291	3.59	0.129	0.018	0.239	0.032	3
J Thorac Imaging	1986	1421	5795	4.08	0.096	0.072	0.121	<0.0010	1
J Ultrasound Med	1982	5488	24732	4.51	0.066	0.059	0.073	<0.0010	3
JBR-BTR	1999	1425	5364	3.76	0.024	-0.007	0.055	0.115	1
Korean J Radiol	2000	1146	6820	5.95	-0.003	-0.059	0.053	0.906	1
Magn Reson Imaging	1985	4439	21690	4.89	0.056	0.043	0.069	<0.0010	2
Magn Reson Imaging C	1994	957	2292	2.39	0.049	0.029	0.07	<0.0010	2
Magn Reson Med	1985	8054	38397	4.77	0.07	0.064	0.076	<0.0010	1
Magn Reson Med Sci	2003	384	2284	5.95	0.061	-0.02	0.142	0.123	1
Neuroimaging Clin N	1995	866	2195	2.54	0.011	-0.02	0.041	0.474	2
Neuroradiology	1972	5281	25145	4.76	0.099	0.091	0.106	<0.0010	2
Pediatr Radiol	1974	6681	25929	3.88	0.039	0.03	0.049	<0.0010	2
Radiographics	1986	3218	14303	4.44	0.052	0.039	0.065	<0.0010	1
Radiol Clin North Am	1964	2791	6249	2.24	0.017	0.012	0.021	<0.0010	3
Radiologe	1961	5677	17128	3.02	0.048	0.04	0.057	<0.0010	2
Radiology	1946	29308	127408	4.35	0.09	0.085	0.095	<0.0010	1
ROFO	1976	9113	36599	4.02	0.072	0.054	0.09	<0.0010	3
Semin Musculoskelet	2001	605	1625	2.69	0.081	0.026	0.135	0.007	3
Semin Roentgenol	1972	1467	3021	2.06	0.021	0.014	0.028	<0.0010	3
Semin Ultrasound CT	1989	1390	3636	2.62	0.047	0.028	0.067	<0.0010	3
Skeletal Radiol	1980	4568	19635	4.30	0.076	0.069	0.083	<0.0010	1
Surg Radiol Anat	1987	2217	10373	4.68	0.065	0.052	0.078	<0.0010	2
Ultraschall Med	1983	1977	8502	4.30	0.094	0.077	0.112	<0.0010	3
Ultrason Imaging	1980	619	2145	3.47	0.052	0.037	0.067	<0.0010	2
Ultrasonics	1967	2199	7699	3.50	0.045	0.033	0.057	<0.0010	2
Ultrasound Med Biol	1975	4916	22609	4.60	0.084	0.076	0.092	<0.0010	2
Ultrasound Obstet Gy	1992	4154	20627	4.97	0.117	0.102	0.132	<0.0010	3
Ultrasound Q	2002	510	1835	3.60	0.153	0.028	0.277	0.021	1

99.6% (216,271/217,142) of articles from 1946–2013 (68 years) were analyzed for overall mean number of authors/article as well as mean number of authors/article per year, categorized by journal, publication type, and language. The remaining 0.04% (871/217,142) of articles were excluded since they had no indexed author information.

**Fig 1:**
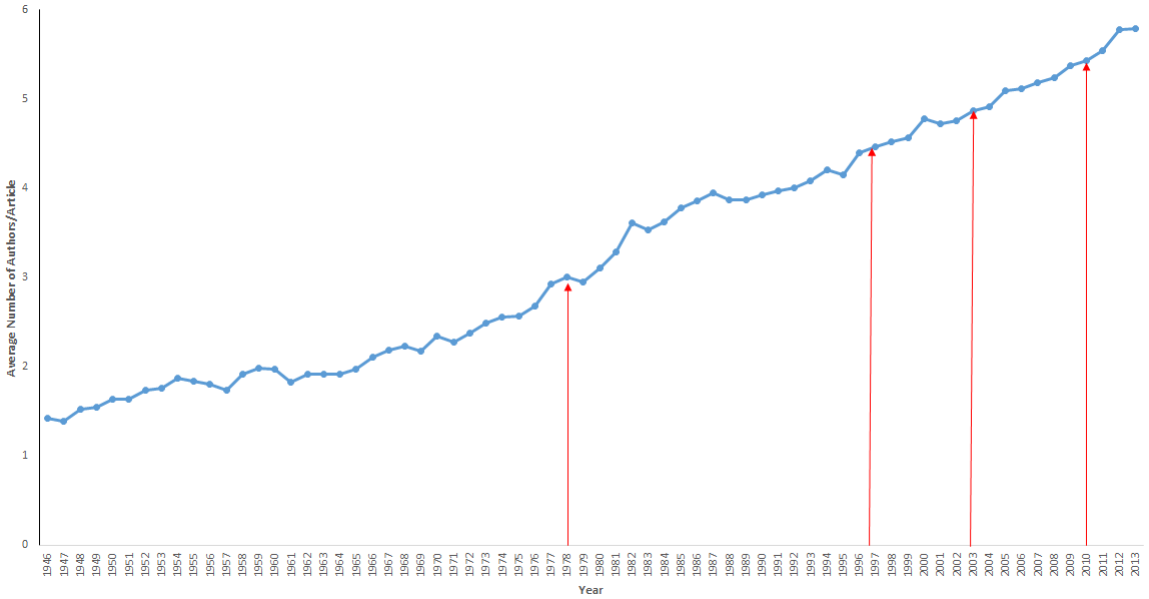
Overall mean number of authors/article for all included journals from 1946 to 2013. Analysis of mean authorship trends over time for radiology journals from 1946 to 2013. Time points for the implementation and major revisions to ICJME guidelines in 1979, 1997, 2003, 2010 and 2013 are denoted by red arrows.

A linear regression analysis of the average number of authors/article over time yielded a proportional relationship between time and mean number of authors/article (Fig 1). In 1946 the mean number of authors per journal was 1.42 and in 2013 it was 5.79. A trend-line demonstrates a consistent increase of 0.07 authors per article per year (R² = 0.9728, P<0.001) from 1946–2013. Visual assessment of the trend line in relation to the time points indicating dissemination of ICMJE guidelines and revisions (Fig 1) documents show no demonstrable impact on the guidelines and the increase in authorship over time.

### Effect of ICJME Guidelines on Authorship

The overall mean number of authors/article was compared in calendar years before and after implementation and revision of ICJME guidelines. The mean number of authors/article was 2.90 in the year before the implementation of ICJME guidelines (1978). This number increased to 2.96 in the year following ICJME implementation (1980), P<0.001. The average authorship in the year (1996) before the first ICJME revision was 4.07. This increased to 4.24 in 1998, P<0001). The mean number of authors/article in the year before the second revision (2009) was 4.95. Authorship increased in the year (2011) following the second ICJME guideline revision to 5.11, P<0.001).

The average number of authors/article in 2013 were compared for 3 groups of journals pertaining to explicit endorsement of ICMJE guidelines on each journal’s website (see Table 1) using a Dunn’s statistical test. The authorship per article was 6.04 for journals that explicitly endorse ICJME guidelines, 4.92 for journals that do not explicitly endorse ICMJE but has a statement regarding authorship criteria, and 4.31 authors/article for journals that have no mention of ICJME or authorship contributions. When the groups are compared, journals that endorse ICJME guidelines have significantly greater number of authors/article than any other journal group (P = 0.03 against journals that do not follow any authorship guidelines and P = 0.02 for journals that do not endorse ICJME but have stated authorship criteria).

### Analysis by Journal

**Fig 2:**
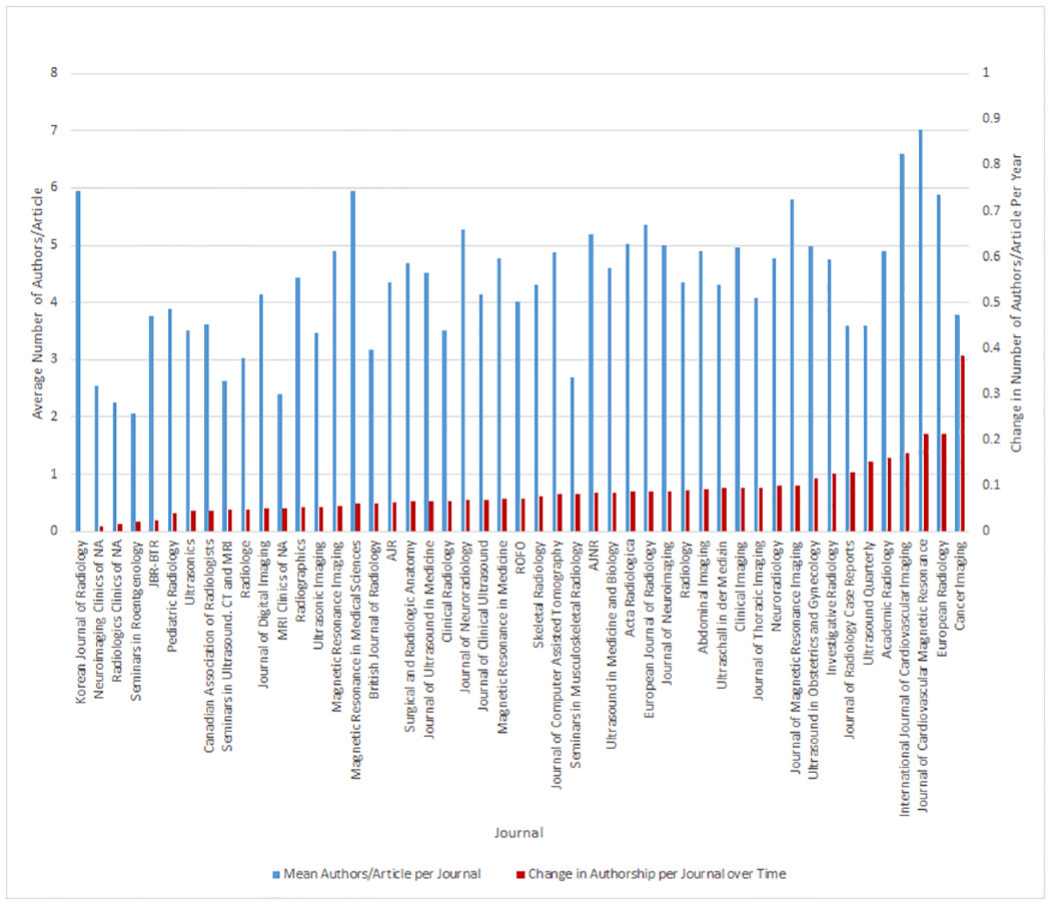
Mean Number of Authors/Article and Change of Authorship per Journal. This is a double axis bar chart showing the mean number of authors/article per journal and the overall change in authorship per journal over time. Journals were organized by change in authorship over time. All journals show an increase in the change in authorship over time with the exception of the *Korean Journal of Radiology* which has a -0.003 change of authorship per year.

The mean number of authors/article and change in authorship over time for each of the 49 journals is presented in Fig 2. The journal with the highest number of authors per article is the *Journal of Cardiovascular Magnetic Resonance* at 7.01, while the journal with the lowest number of authors per article is *Seminars in Roentgenology* at 2.06. The journal with the greatest change of authorship over time (0.384 authors/article per year) was *Cancer Imaging* (R^2^ = 0.871, P<0.001) and the journal with the lowest change of authorship over time was *Korean Journal of Radiology* (-0.003 authors/article per year, P = 0.906). The top 10 journals (*Radiology*, *AJR*, *British Journal of Radiology*, *AJNR*, *ROFO*, *Magnetic Resonance Medicine*, *Clinical Radiology*, *Journal of Computer Assisted Tomography*, *Pediatric Radiology and European Radiology*) by articles published (in descending order) comprise 54% (117,257/217,142) of total articles analyzed (Fig 2).

**Fig 3:**
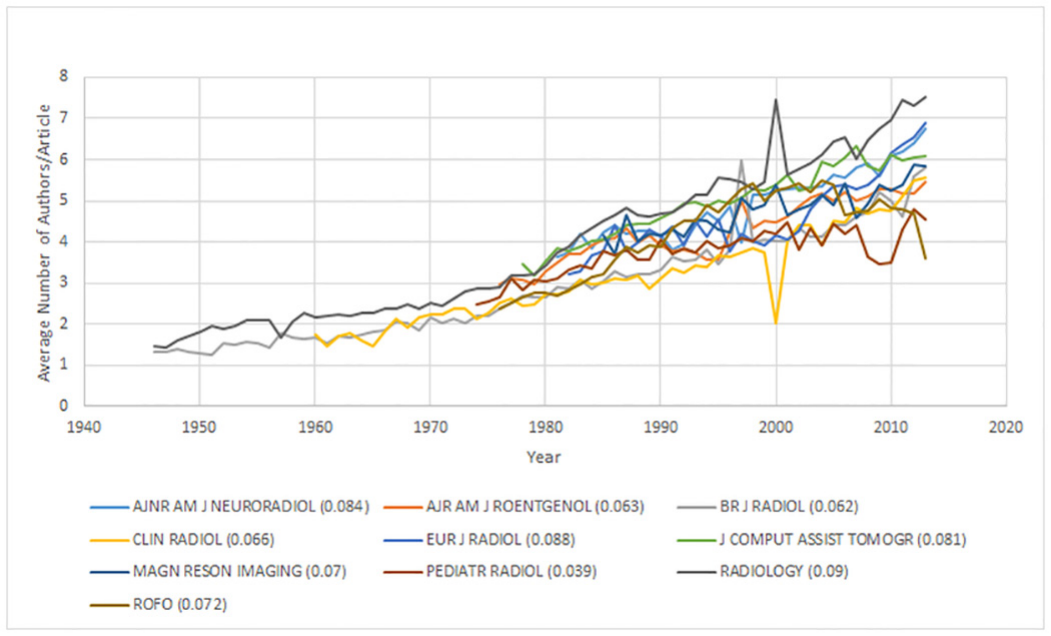
Average Number of Authors/Article per Year for Top 10 Contributing Journals. This graph shows the mean number of authors/article for the Top 10 contributing journals by # of articles published relative to the # of articles published for all radiology journals analyzed.

The majority of these journals (Fig 3) show a strong association between average authorship per article and time (R^2^ ranging from 0.856 to 0.956), while journals, *Pediatric Radiology* and *ROFO*, still show moderate associations (R^2^ of 0.661 and 0.649 for both journals, respectively). Authorship increases range from 0.04–0.213 additional authors per year, P<0.001 for all top 10 journals. Of the top 10 journals, *AJNR* (0.084), *Radiology* (0.09), *ROFO* (0.072), *Magnetic Resonance Medicine* (0.07), *Journal of Computer Assisted Tomography* (0.081) and *European Radiology* (0.213) are above the overall average for change of authorship.

Analysis of all 49 journals shows a range of 2.06–7.01 authors/year. The majority of these journals have a moderate to strong association between authorship and time (R^2^ ranging from 0.512 to 0.956) for the 40 journals analyzed. The remaining 9 journals (*Seminars in Musculoskeletal Radiology*, *Seminars in Roentgenology*, *Ultrasound Quarterly*, *Journal of Digital Imaging*, *Magnetic Resonance Medical Sciences*, *Canadian Association of Radiologists Journal*, *JBR-BTR*, *Neuroimaging Clinics of North America and Korean Journal of Radiology*) have a weak to moderate association (R^2^ ranging from 0.001 to 0.493). These journals are all in the bottom 20 journals by articles published and contribute 4.91% (10,658/217,142) of the articles analyzed. Statistically significant authorship increases range from 0.039–0.384 additional authors per year, P<0.05 for all journals except (*JBR-BTR*, P = 0.115, *Magnetic Resonance Medical Sciences*, P = 0.123, *Neuroimaging Clinics of North America*, P = 0.474, and *Korean Journal of Radiology*, P = 0.906). Only the *Korean Journal of Radiology* has a decrease in change of authorship per year; however this result is statistically insignificant (-0.003 authors per year).

Analysis of journals subcategorized into subspecialty topics (education/review journals, abdominal, ultrasound, CT, MRI, general radiology, neuroradiology, MSK radiology, pediatrics, cancer, thoracic, cardiovascular and miscellaneous) demonstrated that there was no significant difference in change of authorship between different radiology journal subspecialties (P = 0.234).

### Analysis by Primary Author’s Institution Country

51 countries of author origin were identified through Excel coding analysis. Authors from these countries represent 169,171/217,142 articles from 1987–2013. Countries that could not be captured by our analysis were not included in our analysis.

92.3% (156,082/169,171) of all articles from 1987–2013 were classified by the country of origin of the article’s primary author. The remaining articles were not included in our analysis as they could not be categorized due to inconsistent institution reporting standards by MEDLINE in years prior to 1994, an incomplete country list used in coding and lack of feasibility of a manual search due to the number of articles.

**Fig 4:**
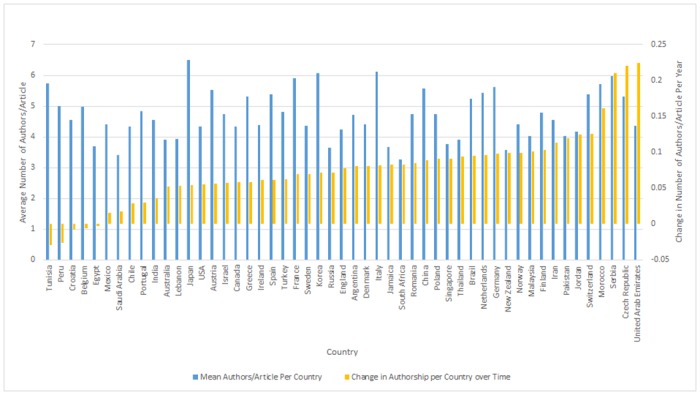
Mean Number of Authors/Article and Change of Authorship per Country. This is a double axis bar chart showing the mean number of authors/article per journal and the overall change in authorship per country over time. Countries were organized by change in authorship over time. Most countries analyzed show an increase in authorship over time with the exception of Tunisia (-0.029), Peru (-0.026), Croatia (-0.008), Belgium (-0.006) and Egypt (-0.003).

The mean number of authors per article and authorship change over time on a per country basis is presented in Fig 4. The country with the highest number of authors per article was Japan at 6.50 and the lowest was South Africa at 3.27. The authorship change was highest for United Arab Emirates (0.535 authors/article per year) and lowest for Tunisia (0.022).

Analyses of all 51 countries shows a range of authorship from 3.27 to 6.50 authors/article. Linear regression analyses show that increases in the mean number of authors/article over time range from 0–0.95. The majority of countries have an increasing authorship over time (0.016–0.225). Egypt, Belgium, Croatia, Peru and Tunisia have a decreasing authorship trend, but for all five of these countries the trend was not significant (p>0.49).

### Analysis by Publication Type

**Fig 5:**
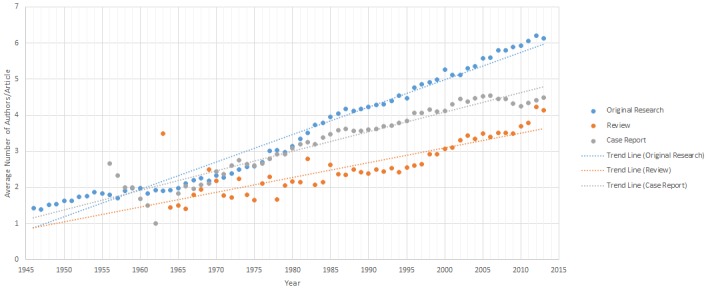
Mean Number of Authors/Article from 1946–2013 by Publication Type. This graph shows the mean number of authors/article for the three publication types analyzed: 1) Case Reports, 2) Reviews and 3) Original Research.

Publication Type analysis was completed for all 217,142 articles. 19,391/217,142 were categorized as “Review articles”, 44,607/217,142 as “Case Reports” and 153,144/217,142 as “Original Research” articles. The mean number of authors/article per year (Fig 5) and linear regression analyses showed increasing authorship per year for all three article types. The overall average number of authors/article for each article type is 4.71 (Original Research), 3.25 (Review), and 3.86 (Case Report) authors per year. With an increase in authors/article per year of 0.076, 0.041 and 0.054 authors/article per year respectively. For all three article types, the number of authors/article per year increased over time, with the greatest increase seen in “Original Research”, followed by “Case Reports” and lastly “Review” articles, P<0.001 for all article types.

### Analysis by Language

**Fig 6:**
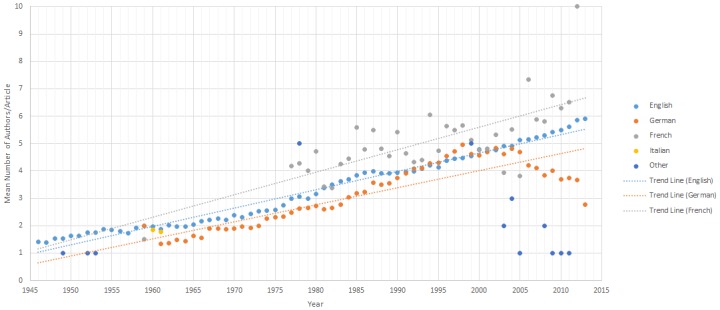
Average Authorship from 1946–2013 by Publication Language. * Trendline for Italian language journals was not included due to data being available for only 2 publication years.

Analysis by publication language was completed for 99.9% (217,077/217,142) articles. 0.1% or 65/217,077 were excluded due to missing data on language of publication. 200,958/217,142 were categorized as “English”, 15,136/217,077 as “German”, 930/217,077 as “French” articles, 39/217,088 as “Italian”, and 14/217,142 as other languages of origin. The change of authorship per year (Fig 6) and linear regression analyses showed increasing authorship per year for all languages of publication. The overall average number of authors/article for each article type is 4.46 (English), 3.61 (German), 4.86 (French), 1.82 (Italian) and 1.86 (Other languages) authors per year. For all major publication languages, the authorship per year increases over time, with the greatest increase seen in “French”, followed by “English” and lastly “German” articles, P<0.001 for all three of these languages. Italian and other publication languages showed no significant increases in authorship; however sample sizes were minor. A regression analysis for publications in Italian could not be completed due to the small range of Italian articles published in time (Table 2).

**Table 2:** Per Publication Language Linear Regression Analysis of Change of Authorship over Time.

**Language**	**Change in Authorship Per Year**	**P-Value**
**English**	0.067	<0.001
**French**	0.082	<0.001
**German**	0.062	<0.001
**Italian**	-0.08	Undefined
**Other**	0.007	0.736

### Relationship between Impact Factor and Average Number of Authors/Article

**Fig 7:**
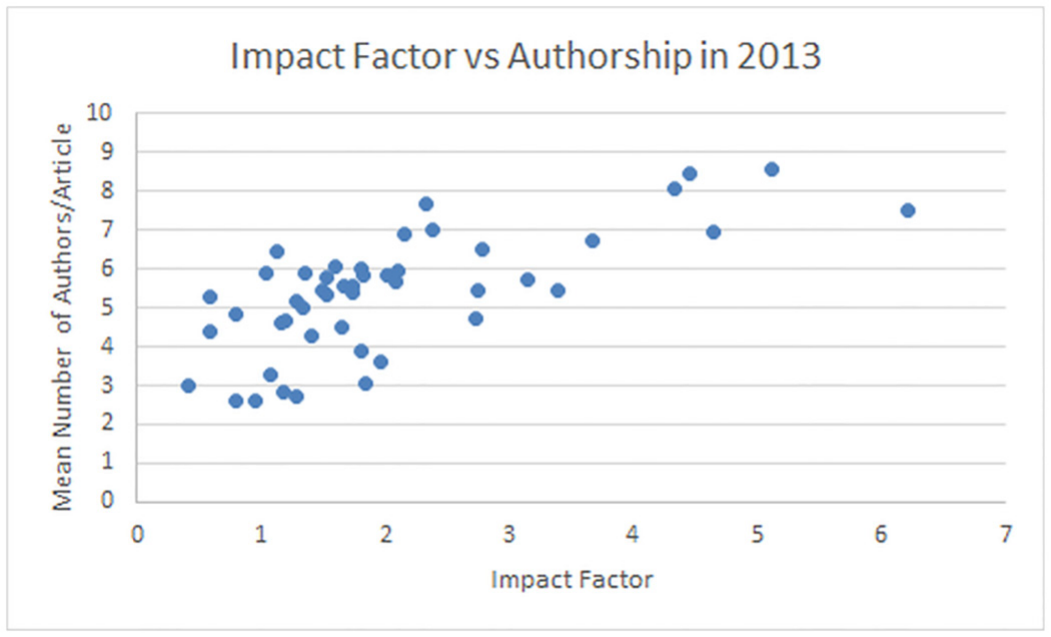
Relationship between Impact Factor and Average Authorship in 2013. The 2013 impact factor of journals analyzed compared against the average number of authors/article in 2013. *JBR-TBR* and *Journal of Radiology Case Reports* were excluded from this graph as they did not have a 2013 impact factor.

A Pearson’s correlation between average authorship and impact factors for 47 journals published in 2013 (see Fig 7), showed a perfect positive association (R = 1, P<0.001). 2 journals were excluded from analysis (*Journal of Radiology Case Reports*, *JBR-BTR*) since they did not have a published impact factor that year.

## Discussion

The overall authorship for 49 radiology journals across 68 years has increased with time. This trend persists independent of the journal of publication, country of publication, publication type and language of publication. However, it is noted, that from the top 10 radiology journals studied, there is an unexplained deviation from this trend, as there was a significant increase in authors/article for *Radiology* and a significant decrease in authors/article for *Clinical Radiology* in the year 2000.

This trend of increasing authorship is consistent with a recent bibliometric analysis by Chow et al (2015). Chow found that the mean number of authors per paper displayed linear growth worldwide from 3.9 to 5.7 (P<0.001) while the number of single authors per paper has decreased by approximately 6.6% from 1991 to 2012. Their analysis concurred with our results showing that there is a significant association between journal impact factor and number of authors per article. Our analysis differs from Chow in that they analyzed any papers where the first author had a radiology department affiliation whereas we investigated radiology journals [6]. Our analysis also includes publications from multiple languages compared to their English-only approach, and investigated the growth of authorship from the implementation of ICJME guidelines and all years of revision. Nonetheless, our study confirms and strengthens their conclusions, as we explore 45 additional years prior to their analysis and 2 years forward—providing a much larger cohort and adding approximately 74,501 articles to this analysis.

Our analysis also shows that publications from all but 5 countries analyzed (Belgium, Croatia, Egypt, Peru, Tunisia) have increasingly more authors per article published per year. In addition, of the Top 10 publishing countries across the timeframe studied, the number of authors per publication and increases in authorship were found to be specifically greater for Asian and European countries compared to publications that have originated from North America (5.67, 5.20 and 4.24 authors/article, respectively). Eisenberg (2014) found that authors from these continents reported a substantially higher prevalence of having a section or department head automatically listed as a coauthor [14]. Our results are uniform with research by Chow (2015) and Hwang (2002) which also demonstrate significantly higher authors/article among primary authors from Asia and Europe compared to authors from North America [6, 30]. These articles also note that increased authorship was correlated with increased perceived honorary authorship. Our study builds on these analyses by exploring authorship contribution for a larger number of articles and for individual countries within each continent.

We also observed that most often the average number of authors/article has increased regardless of the language of publication and publication type. For publication type, paradoxically the number of authors/article is lowest for review articles compared to original research and case reports (4.71 original research, 3.25 review, and 3.86 case report authors per year). In addition, there is a greater increase in authors/article per year for original research of 0.076, compared to 0.054 authors/article per year for review articles, respectively. Given the perceived need for more authors and collaboration for review articles, this result is, perhaps, unexpected.

The average authorship per journal is highly correlated with the impact factor of the journal; the greater the impact factor, the greater the average number of authors per article (R = 1, P<0.001). Four of the five journals with the highest impact factor (*Radiology*, *Journal of Cardiovascular Magnetic Resonance Imaging*, *Investigative Radiology*, and *European Radiology*) also have the highest average number of authors/article. The fifth journal of highest impact factor included in our study, *Ultraschall In Der Medizin*, also has one of the highest authors/article—only the *International Journal of Cardiovascular Imaging*, *Neuroradiology* and the *European Journal of Radiology* had lower impact factors and had higher but similar average number of authors/article (6.75 versus 7.71, 6.99 and 6.89 authors/article respectively). The greatest difference in impact factor and average number of authors/article is observed when journals have an impact factor greater than 4. These journals have an average number of authors/article that range from 6.75–8.59, while all other journals vary significantly from 2.59–7.71 authors/article (see Fig 7).

Our results are also consistent with a study on the effects of author contribution disclosures on authorship trends by McDonald et al (2010). McDonald’s study investigated authorship over time for 16 major medical journals that included *Radiology*, *AJR* and *AJNR* for a 20 year period from 1986–2006. Similar to our study, McDonald demonstrated that journals which did not have authorship limitation policies compared to journals with authorship policies did not slow the trend of increasing numbers of authors per articles over time. The overall number of authors/article over his 20 year analysis of all 16 journals in his study demonstrated increasing authorship at 0.076 authors/year. Our study uses a different methodology of data extraction and expands on McDonald’s analysis by investigating a greater timeframe of 68 years with a particular focus on radiology journals. However, despite the differences between our studies, McDonald’s findings for all high impact factor journals were similar to our study on radiology journals alone, as our overall 0.07 authors/article over time is comparable [28].

The results of our analysis is impactful to the understanding of the prevalence of honorary authorship. Undeserved authorship is a significant issue in high impact factor biomedical journals [31] and is particularly prevalent in major radiology journals with approximately 24–27% of first authors stating that at least one listed author on published papers have insignificant contributions [8, 14]. Only 68% of authors fulfilled ICJME authorship contributions in *Radiology* from 1998–2000 with a significant difference in fulfillment depending on order of authorship with 98.9% and 85.3% of first and second authors fulfilling ICJME criteria compared with 52.8% and 66.5% of fulfillment by middle and last authors respectively [12]. The prevalence of honorary and ghost authorship coincides with increases in frequency of co-authorship. Particularly, an investigation on the *American Journal of Roentgenology* demonstrated a correlation between decreases in authorship contribution with increased number of coauthors within a publication. In this investigation, >87% of articles with more than 5 authors were found to have at least one author who contributed less than 5% to the publication and at least half of those articles had one undeserved coauthor [11]. The average number of authors/paper was 5.79 in 2013. Our investigation explores the trends of radiology co-authorship comprehensively. We have shown that there is a constant increase in authorship in radiology journals over time despite the implementation and revisions of ICJME guidelines.

The causes of increased authorship and possible undeserved authorship is highly speculated and debated. Pressures to publish for academic advancement has been correlated to increases in authorship in academic institutions [13, 25]. Specific academic environments are encouraged to add departmental chairs as authors. This culture remains particularly acceptable in Japan and Germany [32, 33]. Authorship can be also affected by a number of factors in addition to honorary authorship. As diagnostic imaging research increases in complexity, there is the need for more multidisciplinary/multi-institutional collaboration with statistical/ basic science collaboration which may influence the number of authors per article [10, 25, 33]. This hypothesis is supported by the finding that the change in authorship per year is larger for “original research” than for “case reports” and “review” articles.

Our study could be improved by a number of factors. First, our research may be limited by a select list of ‘clinical’ radiology journals that may not be representative of radiology research as a whole. Our list also did not include many new radiology journals that may not be indexed onto MEDLINE. This is particularly true of open access journals such as, *European Journal of Radiology Open* and the *Open Journal of Radiology*. Given that we selected radiology articles exclusively from radiology journals, many landmark radiology research articles in high impact factor, non-radiology journals such as *JAMA* and *New England Journal of Medicine*, were not included in our study. Despite this, our journal list is extensive, encompassing 49 different radiology journals of varying specialities, and includes many of the radiology journals that are well cited and have been in publication for several decades. There are also limits to the methodology of this analysis. Due to the computerized nature of our data extraction, only the primary author could be extracted from each published article. Thus, countries of origin from multi-national studies could not be accounted for. In addition, articles that did not list full author names and published under a research group name were only counted as a single author. Our subtype analyses also could not further classify articles by additional article types. More specifically, less than 5% of article types could be further classified into categories: Observational study, clinical trial, randomized control trial etc.

Although the growth of authorship and undeserved authorship has been observed and acknowledged, there has been a sparse amount of research to clearly elucidate the reasons behind honorary authorship and effective solutions to reduce undeserved authorship has not been explored. As adherence to ICJME guidelines in radiology journals continues to be low [29] and authorship numbers climb despite awareness on undeserved authorship, future research should explore measures that would allow for fair credit for allocated authorship.

## Supporting Information

S1 Table List of included journals. (DOCX)

S2 Table Publication Type of Article Types Selected from MEDLINE. (DOCX)

S3 Table Legend for countries. (DOCX)

S4 Table Number of Articles Excluded Articles by Year. (DOCX)

S1 Appendix Publication Type of Article Types Selected from MEDLINE. (DOCX)

S2 Appendix Coding and Sample Excel Code. (DOCX)
