# Correction: A Comprehensive Analysis of Authorship in Radiology Journals

**DOI:** 10.1371/journal.pone.0147166

**Published:** 2016-01-11

**Authors:** Wilfred Dang, Matthew D. F. McInnes, Ania Z. Kielar, Jiho Hong

There are errors in the published article. The first sentence of the subsection Relationship between Impact Factor and Average Number of Authors/Article in the Results section is incorrect. This sentence should read as follows: “A Pearson’s correlation between average authorship and impact factors for 47 journals published in 2013 (see Fig 7), showed a positive association (R = 0.65, P<0.001)”

The R value in the discussion should read R = 0.65.
