# Supplementary material for: A Comprehensive Analysis of Authorship in Radiology Journals
Source: PLoS One. 2015 Sep 25;10(9):e0139005. doi: 10.1371/journal.pone.0139005 (PMC4583466; doi:10.1371/journal.pone.0139005)
Supplement: S1 Table — (DOCX) [file pone.0139005.s001.docx]

**S1 Table. List of included journals**.

| **ABBREVIATION** | **FULL JOURNAL NAME** |
| --- | --- |
| ABDOM IMAGING | Abdominal Imaging |
| ACAD RADIOL | Academic Radiology |
| ACTA RADIOL | Acta Radiologica |
| AJNR AM J NEURORADIOL | American Journal Of Neuroradiology |
| AJR AM J ROENTGENOL | American Journal Of Roentgenology |
| BR J RADIOL | The British Journal Of Radiology |
| CAN ASSOC RADIOL J | Canadian Association Of Radiology Journal |
| CANCER IMAGING | Cancer Imaging |
| CLIN IMAGING | Clincal Imaging |
| CLIN RADIOL | Clinical Radiology |
| EUR J RADIOL | European Journal Of Radiology |
| EUR RADIOL | European Radiology |
| INT J CARDIOVAS IMAGING | The International Journal Of Cardiovascular Imaging |
| INVEST RADIOL | Investigative Radiology |
| J CARDIOVASC MAGN RESON | Journal Of Cardiovascular Magnetic Resonance |
| J CLIN ULTRASOUND | Journal Of Clinical Ultrasound |
| J COMPUT ASSIST TOMOGR | Journal Of Computer Assisted Tomography |
| J DIGIT IMAGING | Journal Of Digital Imaging |
| J MAGN RESON IMAGING | Journal Of Magnetic Resonance Imaging |
| J NEUROIMAGING | Journal Of Neuroimaging |
| J NEURORADIOL | Journal Of Neuroradiology |
| J RADIOL CASE REPORTS | Journal Of Radiology Case Reports |
| J THORAC IMAGING | Journal Of Thoracic Imaging |
| J ULTRASOUND MED | Journal Of Ultrasound Medicine |
| JBR-BTR | Journal Belge De Radiologie-Belgisch Tijdschrift Voor Radiologie |
| KOREAN J RADIOL | Korean Journal Of Radiology |
| MAGN RESON IMAGING | Magnetic Resonance Imaging |
| MAGN RESON IMAGING CLIN N AM | Magnetic Resonance Imaging Clinics Of North America |
| MAGN RESON MED | Magnetic Resonance In Medicine |
| MAGN RESON MED SCI | Magnetic Resonance In Medical Sciences |
| NEUROIMAGING CLIN N AM | Neuroimaging Clinics Of North America |
| NEURORADIOLOGY | Neuroradiology |
| PEDIATR RADIOL | Pediatric Radiology |
| RADIOGRAPHICS | Radiographics |
| RADIOL CLIN NORTH AM | Radiologic Clinics Of North America |
| RADIOLOGE | Radiologe |
| RADIOLOGY | Radiology |
| ROFO | Fortschritte Auf Dem Gebiet Der Röntgenstrahlen Und Der Bildgebenden Verfahren |
| SEMIN MUSCULOSKELET RADIOL | Seminars In Musculoskeletal Radiology |
| SEMIN ROENTGENOL | Seminars In Roentgenology |
| SEMIN ULTRASOUND CT MR | Seminars In Ultrasound, Ct, And Mri |
| SKELETAL RADIOL | Skeletal Radiology |
| SURG RADIOL ANAT | Surgical And Radiologic Anatomy |
| ULTRASCHALL MED | Ultraschall In Der Medizin |
| ULTRASON IMAGING | Ultrasound Imaging |
| ULTRASONICS | Ultrasonics |
| ULTRASOUND MED BIOL | Ultrasound In Medicine And Biology |
| ULTRASOUND OBSTET GYNECOL | Ultrasound In Obstetrics And Gynecology |
| ULTRASOUND Q | Ultrasound Quarterly |
