# Supplementary material for: A Comprehensive Analysis of Authorship in Radiology Journals
Source: PLoS One. 2015 Sep 25;10(9):e0139005. doi: 10.1371/journal.pone.0139005 (PMC4583466; doi:10.1371/journal.pone.0139005)
Supplement: S2 Table — (DOCX) [file pone.0139005.s002.docx]

**S3 Table. Publication Type of Article Types Selected from MEDLINE:**

| **MEDLINE PUBLICATION TYPE** |
| --- |
| Case Reports |
| Classical Article |
| Clinical Trial, All |
| Clinical Trial, Phase I |
| Clinical Trial, Phase II |
| Clinical Trial, Phase III |
| Clinical Trial, Phase IV |
| Clinical Trial |
| Comparative Study |
| Controlled Clinical Trial |
| Evaluation Studies |
| Guideline |
| In Vitro |
| Introductory Journal Article |
| Journal Article |
| Meta Analyses |
| Multicenter Study |
| Observational Study |
| Pragmatic Clinical Trial |
| Practice Guideline |
| Randomized Controlled Trial |
| Research Support, American Recovery and Reinvestment Act |
| Research Support, NIH, Extramural |
| Research Support, NIH, Intramural |
| Research Support, Non US Gov’t |
| Research Support, US Gov’t, Non PHS |
| Research Support, US Gov’t, PHS |
| Review |
| Scientific Integrity Review |
| Systematic Reviews |
| Technical Report |
| Twin Study |
| Validation Studies |
