# Supplementary material for: A Comprehensive Analysis of Authorship in Radiology Journals
Source: PLoS One. 2015 Sep 25;10(9):e0139005. doi: 10.1371/journal.pone.0139005 (PMC4583466; doi:10.1371/journal.pone.0139005)
Supplement: S3 Table — (DOCX) [file pone.0139005.s003.docx]

**S4 Table. Legend for countries**

| **CODE** | **COUNTRY** |
| --- | --- |
| 0 | No Country |
| 1 | New Zealand |
| 2 | Spain |
| 3 | Canada |
| 4 | China (including Hong Kong and Taiwan) |
| 5 | Korea |
| 6 | Japan |
| 7 | France |
| 8 | Greece |
| 9 | Italy |
| 10 | Germany |
| 11 | England/United Kingdom/Britain |
| 12 | Turkey |
| 13 | Saudi Arabia |
| 14 | India |
| 15 | Switzerland |
| 16 | Egypt |
| 17 | Ireland |
| 18 | Finland |
| 19 | Sweden |
| 20 | South Africa |
| 21 | Australia |
| 22 | Israel |
| 23 | Denmark |
| 24 | Netherlands |
| 25 | Brazil |
| 26 | Portugal |
| 27 | Austria |
| 28 | Belgium |
| 29 | Thailand |
| 30 | Russia |
| 31 | Norway |
| 32 | Czech Republic |
| 33 | Argentina |
| 34 | Croatia |
| 35 | Jordan |
| 36 | Iran |
| 37 | Singapore |
| 38 | Poland |
| 39 | Chile |
| 40 | Romania |
| 41 | USA |
| 42 | Malayasia |
| 43 | Lebanon |
| 44 | Serbia |
| 45 | Mexico |
| 46 | Morocco |
| 47 | Tunisia |
| 48 | Jamaica |
| 49 | Peru |
| 50 | United Arab Emirates |
| 51 | Pakistan |
