# Supplementary material for: A Comprehensive Analysis of Authorship in Radiology Journals
Source: PLoS One. 2015 Sep 25;10(9):e0139005. doi: 10.1371/journal.pone.0139005 (PMC4583466; doi:10.1371/journal.pone.0139005)
Supplement: S4 Table — (DOCX) [file pone.0139005.s004.docx]

**S5 Table. Number of Articles Excluded Articles by Year**

| **YEAR** | **NUMBER OF ARTICLES EXCLUDED** |
| --- | --- |
| 1946 | 0 |
| 1947 | 1 |
| 1948 | 0 |
| 1949 | 0 |
| 1950 | 3 |
| 1951 | 8 |
| 1952 | 3 |
| 1953 | 6 |
| 1954 | 3 |
| 1955 | 3 |
| 1956 | 6 |
| 1957 | 3 |
| 1958 | 3 |
| 1959 | 1 |
| 1960 | 2 |
| 1961 | 1 |
| 1962 | 0 |
| 1963 | 1 |
| 1964 | 3 |
| 1965 | 2 |
| 1966 | 5 |
| 1967 | 3 |
| 1968 | 2 |
| 1969 | 5 |
| 1970 | 1 |
| 1971 | 4 |
| 1972 | 7 |
| 1973 | 10 |
| 1974 | 5 |
| 1975 | 4 |
| 1976 | 10 |
| 1977 | 18 |
| 1978 | 12 |
| 1979 | 18 |
| 1980 | 15 |
| 1981 | 15 |
| 1982 | 16 |
| 1983 | 25 |
| 1984 | 25 |
| 1985 | 23 |
| 1986 | 36 |
| 1987 | 39 |
| 1988 | 59 |
| 1989 | 55 |
| 1990 | 57 |
| 1991 | 15 |
| 1992 | 23 |
| 1993 | 12 |
| 1994 | 9 |
| 1995 | 17 |
| 1996 | 16 |
| 1997 | 40 |
| 1998 | 29 |
| 1999 | 31 |
| 2000 | 12 |
| 2001 | 8 |
| 2002 | 11 |
| 2003 | 7 |
| 2004 | 11 |
| 2005 | 35 |
| 2006 | 8 |
| 2007 | 2 |
| 2008 | 6 |
| 2009 | 18 |
| 2010 | 6 |
| 2011 | 3 |
| 2012 | 9 |
| 2013 | 25 |
| TOTAL | 871 |
