# Supplementary material for: A Comprehensive Analysis of Authorship in Radiology Journals
Source: PLoS One. 2015 Sep 25;10(9):e0139005. doi: 10.1371/journal.pone.0139005 (PMC4583466; doi:10.1371/journal.pone.0139005)
Supplement: S1 Appendix — (DOCX) [file pone.0139005.s005.docx]

**S2 Appendix.** **Publication Type of Article Types Selected from MEDLINE**

Categories of journal for comparison

**EDUCATION/REVIEW JOURNALS (not original research)**

ULTRASOUND Q

MAGN RESON IMAGING CLIN N AM

RADIOGRAPHICS

RADIOL CLIN NORTH AM

SEMIN ULTRASOUND CT MR

SEMIN ROENTGENOL

NEUROIMAGING CLIN N AM

SEMIN MUSCULOSKELET RADIOL

**Abdominal**

ABDOM IMAGING

**Ultrasound**

J CLIN ULTRASOUND

J ULTRASOUND MED

ULTRASCHALL MED

ULTRASON IMAGING

ULTRASONICS

ULTRASOUND MED BIOL

ULTRASOUND OBSTET GYNECOL

ULTRASOUND Q

**SEMIN ULTRASOUND CT MR**

**CT**

J COMPUT ASSIST TOMOGR

SEMIN ULTRASOUND CT MR

**MRI**

J CARDIOVASC MAGN RESON

J MAGN RESON IMAGING

MAGN RESON IMAGING

MAGN RESON IMAGING CLIN N AM

MAGN RESON MED

MAGN RESON MED SCI

SEMIN ULTRASOUND CT MR

**General Radiology**

ACAD RADIOL

Acta Radiologica

AJR

Br J RADIOL

CAN ASSOC RADIOL J

CLIN IMAGING

CLIN RADIOL

EUR J RADIOL

EUR RADIOL

INVEST RADIOL

J RADIOL

JBR-BTR

KOREAN J RADIOL

RADIOLOGE
RADIOLOGY

ROFO

SEMIN ROENTGENOL

SEMIN ULTRASOUND CT MR

**Neuroradiology**

AJNR

J NEUROIMAGING

J NEURORADIOL

NEUROIMAGING CLIN N AM

NEURORADIOLOGY

**MSK Radiology**

SEMIN MUSCULOSKELET RADIOL

SKELETAL RADIOL

**Pediatrics**

PEDIATR RADIOL

**Cancer**

CANCER IMAGING

**Thoracic**

J THORAC IMAGING

**Cardiovascular**

INT J CARDIOVASC IMAGING

J CARDIOVASC MAGN RESON

**Misc**

J DIGIT IMAGING

SURG RADIOL ANAT
