# Supplementary material for: A Comprehensive Analysis of Authorship in Radiology Journals
Source: PLoS One. 2015 Sep 25;10(9):e0139005. doi: 10.1371/journal.pone.0139005 (PMC4583466; doi:10.1371/journal.pone.0139005)
Supplement: S2 Appendix — (DOCX) [file pone.0139005.s006.docx]

**S6 Appendix. Coding and Sample Excel Code**

Authors

*=IF(ISEVEN(LEN(AUTHORS_CELL)-LEN(SUBSTITUTE(AUTHORS_CELL,CHAR(10),""))+1),(LEN(AUTHORS_CELL)-LEN(SUBSTITUTE(AUTHORS_CELL,CHAR(10),""))+1)/2,(LEN(AUTHORS_CELL)-LEN(SUBSTITUTE(AUTHORS_CELL,CHAR(10),""))+2)/2)*

Country

*=IF((ISNUMBER(SEARCH("New Zealand",INSTITUTION_CELL))),1,IF((ISNUMBER(SEARCH("Spain", INSTITUTION_CELL))),2,IF((ISNUMBER(SEARCH("Canada", INSTITUTION_CELL))),3,IF((ISNUMBER(SEARCH("China", INSTITUTION_CELL))),4,IF((ISNUMBER(SEARCH("Korea", INSTITUTION_CELL))),5,IF((ISNUMBER(SEARCH("Japan", INSTITUTION_CELL))),6,"N/A"))))))*

*=IF(ISNUMBER(SEARCH(", AL ",INSTITUTION_CELL)),"USA",IF(ISNUMBER(SEARCH(", AK ", INSTITUTION_CELL)),"USA",IF(ISNUMBER(SEARCH(", AZ ", INSTITUTION_CELL)),"USA",IF(ISNUMBER(SEARCH(", AR ", INSTITUTION_CELL)),"USA",IF(ISNUMBER(SEARCH(", CA ", INSTITUTION_CELL)),"USA",IF(ISNUMBER(SEARCH(", CO ", INSTITUTION_CELL)),"USA","N/A"))))))*

Language

*=IF(ISNUMBER(SEARCH("Italian",LANGUAGE)),"Italian",IF(ISNUMBER(SEARCH("French", LANGUAGE)),"French",IF(ISNUMBER(SEARCH("German", LANGUAGE)),"German",IF(ISNUMBER(SEARCH("English", LANGUAGE)),"English","Other"))))*

Publication Type

*=IF(ISNUMBER(SEARCH("Case Report", PUBLICATION_TYPE)),2,IF(ISNUMBER(SEARCH("Review", PUBLICATION_TYPE)),1,IF(ISNUMBER(SEARCH("Journal Article", PUBLICATION_TYPE)),0,3)))*
